# Confirmatory Person-Oriented Mediation Analysis in Categorical Variables-Options and Limitations

**DOI:** 10.1007/s12124-026-10023-2

**Published:** 2026-08-10

**Authors:** Alexander von Eye, Wolfgang Wiedermann

**Affiliations:** 1 Michigan State University, East Lansing, MI USA; 2 University of Missouri, Columbia, USA

**Keywords:** Full mediation, Partial mediation, Statement calculus, Relevance logic, Configural frequency analysis

## Abstract

In this article, we propose three new approaches to analyzing person-oriented mediation hypotheses. First, we propose using relevance logic instead of classical logic. Relevance logic (1) rejects the ex falso sequitur quodlibet (“from a falsehood anything follows”) rule and (2) requires that the premise and the outcome of an implication exhibit variable sharing, that is, possess a substantive link. Propositions are declared irrelevant when the premise is false, when there is no substantive link, or both. Second, we discuss, based on relevance logic, alternative concepts of mediation. Specifically, we discuss that mediation can be based on conjunctive as well as on implicative relations. In contrast to conjunctive mediation models, implicative mediation models do allow one to distinguish between full and partial mediation. Third, we propose using confirmatory Configural Frequency Analysis to test hypotheses of full or partial mediation. This is illustrated using data on adolescent development.

Models of mediation include at least three variables, the predictor, *X*, the mediator, *M*, and the outcome variable, *Y* (Baron & Kenny, 1986; MacKinnon et al., 2007; Wiedermann et al., 2019; Wiedermann & von Eye, 2015; Wiedermann & Eye, 2016; Wright, 1921). The flow of mediation effects originates in *X* and reaches *Y* either by way of *M* or directly. When the paths go *X* → *M* → *Y*, it is termed *full mediation*. When the paths go both, *X* → *M* → *Y* and *X* → *Y*, it is termed *partial mediation*. When the path only goes *X* → *Y*, there is no mediation. Figure 1 displays the three scenarios.

**Fig. 1:**
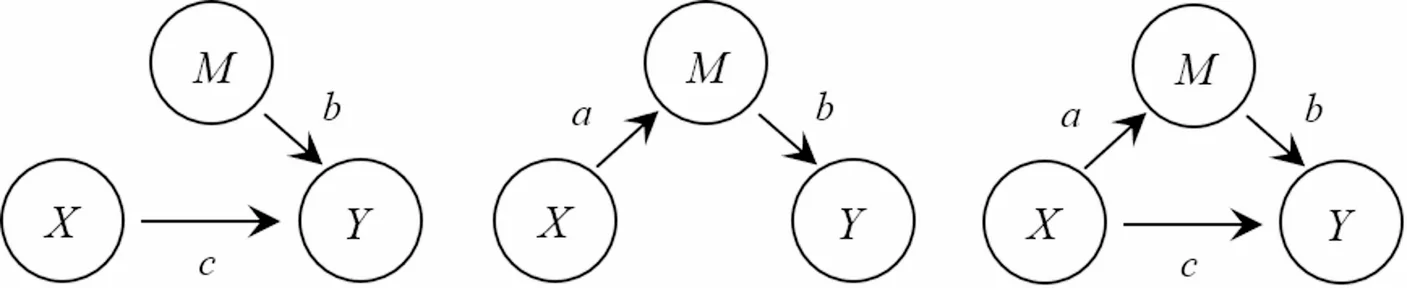
Mediation of the flow from *X* to *Y* by *M*. Left panel: No mediation (*M* serves as a covariate). Middle Panel: Full mediation. Right Panel: Partial mediation

In Fig. 1 (middle panel), paths *a* and *b* jointly represent full mediation. Paths *a*, *b*, and *c* jointly represent partial mediation. When only Path *c* connects *X* and *Y*, a standard two-variable regression model can represent the flow of effects, and there is no mediation. A large body of literature exists that addresses several aspects of statistical mediation analysis. Here, the majority of research focuses on the statistical performance of significance tests to evaluate mediation hypotheses (e.g., MacKinnon et al., 2002; Shrout & Bolger, 2002), counterfactual definitions of causal mediation mechanisms (e.g., Imai et al., 2010; Pearl, 2012; Valeri & VanderWeele, 2013), and the development of statistical methods to evaluate mediation in the context of mixed variable scales (e.g., instances in which one or more variables are measured in categorical form; e.g., Rijnhart et al., 2023). In contrast, sound statistical methods for the evaluation of mediational mechanisms from a person-oriented perspective received less attention (for exceptions, see von Eye et al., 2009; Smyth & MacKinnon, 2020; Wiedermann & von Eye, 2021). The present article continues the discussion of person-oriented mediation analysis and proposes utilizing principles of relevance logic to conduct a confirmatory evaluation of person-oriented mediational mechanisms.

This article is structured as follows. First, we briefly review variable-oriented approaches to mediation analysis. This is followed by review of exploratory categorical-variable approaches to mediation analysis. Issues with the latter approach are discussed in the context of confirmatory research in which statement calculus is used to determine which patterns of variable categories can be viewed as in support of mediation hypotheses. Relevance logic (for an overview, see Mares, 2024) is discussed as a viable approach to specify such patterns, and Configural Frequency Analysis is discussed as a statistical method that can be used to answer the question whether and – if yes – what form of mediation exists in empirical data sets.

## Variable-Oriented Mediation Analysis

The three-variable mediation model of the cause, *X*, the mediator, *M*, and the outcome, *Y*, that was depicted in Fig. 1 can be represented by three regression equations. In the first, Path *M* → *X*, *M* is regressed onto *X*, that is (without loss of generality we assume that all variables are mean-centered)

$$\:M=aX+{\epsilon\:}_{1}$$

where *a* is the regression parameter and *ε*_1_ represents the residuals. In the second equation, the effects of *X* and *M* on *Y* are estimated, or

$$\:Y=cX+bM+{\epsilon\:}_{2}$$

where *c* is the direct effect of *X* on *Y*, *b* is the parameter for the regression of *Y* on *M*, and *ε*_2_ represents the residuals for this regression. The third equation represents the total effect of *X* on *Y*, or

$$\:Y={c}^{{\prime\:}}X+{\epsilon\:}_{3}$$

where $\:{c}^{{\prime\:}}$ is the parameter for the regression of *Y* on *X*, that is, the total effect of *X* on *Y* (without taking into account the mediator, *M*), and *ε*_3_ represents the residuals for this regression. The magnitudes of the indirect and the total effects can be calculated as follows:

$$Indirect\;effect=a\cdot b,\;and$$

$$Total\;effect=c+a\cdot b$$

For the application of these equations, it must be assumed that the relations among *X*, *M*, and *Y* are linear and the dependent variables are continuous (cf. Ledermann et al., 2025; approaches for categorical mediators and outcomes are, for example, presented by Rijnhart et al., 2023). In addition, it must be assumed that the effects are valid over the entire range of scores of *X*, *M*, and *Y*. In the analysis of binary variables, it must be assumed that mediation-specific variable relations are valid for all variable categories (for log-linear mediation models and effect size measures, see Eshima, & Tabata, 1999; Wiedermann, & von Eye, 2021). This applies accordingly when variables have more than two categories.

## Exploratory Person-Oriented Mediation Analysis

In person-oriented (Bergman & Magnusson, 1997; von Eye & Bergman, 2003; von Eye et al., 2015) analysis of categorical variables, the assumption that relations can be observed in all variable categories is relaxed. Instead, it is assumed that relations can be only observed in some categories, but not in others. In exploratory person-oriented research, the category patterns (configurations) that carry relations are not known or hypothesized a priori. Therefore, search algorithms have been developed to identify these patterns.

Exploratory Configural Frequency Analysis (CFA; Lienert, 1968; von Eye, & Gutiérrez Peña, 2004; von Eye, & Wiedermann, 2021) is often seen as the method of choice for this purpose. CFA uses probability models, known as *base models*, that include all effects that are not of interest to the researcher. When these models are rejected, effects of interest are bound to exist. This approach can be viewed as an example of falsificationism (Popper, 1959; Lakatos, 1970). That is, rejection of the base model provides the starting point for CFA to identify those patterns of variable categories that carry the effects that contradict the base model most prominently.

Virtually all existing base models of CFA can be specified using the following model:

$$\:\mathrm{log}\widehat{m}=\left[1\:\mathbf{X}\right]\left[\begin{array}{c}{\lambda\:}_{c}\\\:{\Lambda\:}\end{array}\right]\:\:$$

where $\:\widehat{m}$ is the vector of estimated cell frequencies, **1** is the constant vector, and **X** is the design matrix that contains the vectors for the effects that are not of interest to the researcher. **X** contains, for example, main effects and interactions, vectors that distinguish groups of data carriers, or covariates. λ_*c*_ is the parameter of the model constant and vector Λ contains the parameters of the vectors in **X** (for more detail on CFA base models, see von Eye, & Wiedermann, 2025; von Eye et al., 2026).

There are two ways to use such a CFA base model. The first is exploratory. The entire cross-classification is searched for cells (configurations of variable categories) that contain significantly more (or fewer) cases than expected from the base model. These cells are said to constitute CFA types (or antitypes). The second is confirmatory. A priori specified individual cells or groups of cells are examined. These cells are hypothesized to represent particular effects. In the approach proposed in this article, these cells are specified to reflect hypotheses of mediation.

For exploratory mediation CFA, three approaches have been proposed. In the first (von Eye et al., 2009), a series of models is tested to determine which of the paths *a*, *b*, and *c* in Fig. 1 exist. Depending on the results of these tests, a joint base model is used to identify and interpret the configurations that represent full or partial mediation. In the second approach (Smyth, & MacKinnon, 2020), there is no joint base model. Therefore, the number of CFA models to be tested is larger, and results can be more detailed. In the third approach (Wiedermann, & von Eye, 2021), the search is simplified by way of estimating specific log-linear models quantifying direct and indirect effects before searching for configurations that represent mediation.

These three approaches have in common that they (1) are exploratory and (2) focus on individual configurations that represent either full or partial mediation. In addition, the first two approaches are complex to the extent that they have found limited application. In the following sections, we propose a new approach to configural mediation analysis. This approach is considerably simpler than the three existing approaches. Specifically, this approach.

1. is confirmatory; the configurations of interest, that is, the configurations that are hypothesized to carry a mediation process, are determined before statistical analysis;
2. it uses relevance logic (to be discussed in the next section; for a more detailed overview, see Mares, 2024; Reed, 1988; Standefer, 2024) to identify the configurations of interest; and.
3. it performs statistical tests only on the configurations of interest, thus simplifying analysis.

## Symmetry in Mediation Analysis

Statement calculus, also called propositional logic or zeroth order logic, is concerned with propositions and relations between propositions (see Bauer, & Wirsing, 1991; Franks, 2023). In two-valued logic (in this article, we focus on two-valued logic; for an introduction into three-valued logic, see Kleene, 1952) propositions can be either true or false. Operators are used to connect propositions. The resulting composite propositions are also either true or false (see Boole, 1854; Wittgenstein, 1905). Table 1 displays the truth values for two elementary propositions, *X* and *Y*, and composite propositions that result from connecting two elementary propositions with the four operators *and* (conjunction; ∧), *or* (disjunction; ∨), *implication*, (→), and *reverse implication* (←).

**Table 1:** Truth values {T, F} for the elementary propositions *X* and *Y*, and the four operators *and* (conjunction; ∧), *or* (disjunction; ∨), *implication*, (→), and *reverse implication* (←)

X	Y	X ∧ Y	X ∨ Y	X → Y	X ← Y
T	T	T	T	T	T
T	F	F	T	F	T
F	T	F	T	T	F
F	F	F	F	T	T

The cross-tabulation of the elementary propositions *X* and *Y* allows one to determine the truth of the composite propositions that result from applying the operators. Specifically, a conjunction *X* ∧ *Y* is true only when both, *X* and *Y* are true. A disjunction, *X* ∨ *Y*, is true when one or both of *X* and *Y* are true. The implication, *X* → *Y*, is true when both, *X* and *Y*, are true, or when *X* is false. Accordingly, the reverse implication, *X* ← *Y*, is true when both, *Y* and *X* are true, or when *Y* is false (detailed explanations *why an implication is true when the premise is false will be given later on).*

For the present context, the configural analysis of mediation, most important is the property of *commutativity*. When a logical connector possesses commutativity, the order of elementary propositions can be reversed without change in the truth of the composite proposition. Conjunction and disjunction are commutative or, more specifically:

$$\:X\wedge\:Y\equiv\:Y\wedge\:X$$

where ≡ is the symbol for ‘is identical to’ or ‘is equivalent with,’ and

$$\:X\vee\:Y\equiv\:Y\vee\:X$$

In contrast, the implication is not commutative, or

$$\:X\to\:Y\not\equiv\:Y\leftarrow\:X$$

where ≢ is the symbol for ‘is not identical to’ or ‘is not equivalent with,’ In words, when it is true that *X* implies *Y*, the reverse implication, that is, *Y* implies *X*, is not equivalent. Table 1 displays these four cases. Later in this article, we discuss why operators that are not commutative are of importance in configural mediation analysis.

With respect to standard regression methods used for regression and mediation analysis, we note that they are based on covariances, that is, second order moments (cf., von Eye, & DeShon, 2012; Wiedermann, & von Eye, 2025). Regressing *Y* on *X*, i.e., estimating the linear model $\:Y={a}_{YX}+{b}_{YX}X+{\epsilon\:}_{YX}$, the regression coefficient *b*_*YX*_ is obtained through

$$\:{b}_{YX}=\frac{co{v}_{XY}}{va{r}_{X}}$$

where *cov*_*XY*_ is the covariance of *X* and *Y*, and *var*_*X*_ is the variance of the predictor *X*. In contrast, regressing *X* on *Y*, i.e., entertaining the linear model $\:X={a}_{XY}+{b}_{XY}Y+{\epsilon\:}_{XY}$, the corresponding slope coefficient becomes

$$\:{b}_{XY}=\frac{co{v}_{XY}}{va{r}_{Y}}$$

Clearly, the two coefficients share the covariance of *X* and *Y* as their basis and they are equivalent in the standardized case (i.e., when *var*_*X*_ = *var*_*Y*_ = 1). They are commutative. For standardized variables, one obtains

$$\:{b}_{YX}={b}_{XY}=\frac{co{v}_{XY}}{va{r}_{X}}=\frac{co{v}_{XY}}{va{r}_{Y}}=co{v}_{XY}={\rho\:}_{XY}$$

with $\:{\rho\:}_{XY}$ denoting the Pearson correlation coefficient. Similarly, in the unstandardized case, differences between $\:{b}_{YX}$ and $\:{b}_{XY}$ only depend on the variability in *X* and *Y* and it is straightforward to convert one regression coefficient into the other. Specifically,

$$\:{b}_{YX}={b}_{XY}\left(va{r}_{Y}/va{r}_{x}\right)$$ $$\:{b}_{XY}={b}_{YX}\left(va{r}_{X}/va{r}_{Y}\right)$$

which implies that

$$\:{b}_{YX}\left(s{d}_{X}/s{d}_{Y}\right)={b}_{XY}\left(s{d}_{Y}/s{d}_{X}\right)$$

with *sd*_*X*_ and *sd*_*Y*_ denoting the standard deviations of *X* and *Y*.

This illustrates that regression coefficients, covariances, and correlations are commutative. Therefore, they cannot be used to determine whether an effect originates in *X* or in *Y*. For this reason, it has been proposed using higher order moments for evaluating the causal direction of dependence (see Dodge, & Rousson, 2001; von Eye, & DeShon, 2012; Wiedermann, & von Eye, 2015, 2025) which preserve the fundamental asymmetry of cause and effect.

Similarly, for categorical variables, interactions or associations are symmetric as well. This can be illustrated as follows. Consider the two binary variables *A* and *B*. To represent the *A* × *B* interaction, one performs the element-wise multiplication of the main effect terms of *A* and *B*. Similarly, to represent the *B* × *A* interaction, one performs the element-wise multiplication of the main effect terms of *B* and *A*. As is well known, changing the order of multiplicands has no effect on the result of a multiplication. This is illustrated in Table 2 for the two binary variables *A* and *B*.

**Table 2:** Representation of the *A* × *B* and the *B* × *A* interactions (main effects and interactions given in effect coding)

Main Effect A	Main Effect B	Interaction A × B	Interaction B × A
1	1	1	1
1	–1	–1	–1
–1	1	–1	–1
–1	–1	1	1

Evidently, the last two columns of Table 2 are the same. This can be viewed as parallel to the calculation of the cross-product to obtain the covariance of two variables, correlations, or regression coefficients. There is one consequence of the symmetry property of second central moments and first order interactions, namely, *using measures that are based on second central moments and first order interactions*,* hypotheses cannot be tested that involve direction of causation*.

Several methods have been suggested for addressing this problem. In this section, we briefly examine four of them (see also Wiedermann, & von Eye, 2016; Wiedermann et al., 2021) and continue developing a fifth approach in the context of mediation analysis. The first method is experimental research (see, e.g., Shadish et al., 2002). Because experiment designs generate data under controlled conditions, they make it possible to test directional hypotheses and are therefore often perceived as the “gold standard.” However, some research questions cannot be investigated experimentally due to ethical constraints. Furthermore, in the context of evaluating mediational mechanisms, experimental designs can be of limited use. The reason for this is that often only the predictor (*X*) is under experimental control. Even if the predictor is randomized (i.e., randomly allocating study participants to control and treatment groups), the causal effect of the mediator (*M*) on the outcome (*Y*) is purely observational and, therefore, rests on unconfoundedness and direction of causation assumptions (cf. Wiedermann et al., 2019; Wiedermann & Li, 2020). Although blockage- and enhancement designs have been proposed (Imai et al., 2011) which, in addition to the predictor, experimentally control the hypothesized mediator, these designs also rest on strong assumptions (for details, see Bullock et al., 2010).

A second approach – methods of Direction Dependence Analysis (DDA; Wiedermann & von Eye, 2025) and Linear Non-Gaussian Acyclic Models (LiNGAM; Shimizu et al., 2006) – relies on third- and fourth-order moments. These moments preserve information that can help determine the direction of causation. Although these approach have been extensively developed (see, e.g., Shi et al., 2026), supported by available software (see, e.g., Ikeuchi et al., 2023; Wiedermann & Li, 2018; Wiedermann & Hirni, 2025), and illustrated in the context of mediation analysis through several practical applications (see, e.g., Wiedermann et al., 2020; Wiedermann & Sebastian, 2020), they are, nevertheless, of limited use for the present discussion because DDA and LiNGAM were predominantly designed for continuous variables (for exceptions, see Inazumi et al. 2011; Wiedermann & von Eye, 2020a), whereas the current focus is on categorical variables.

A third method, proposed by von Eye & Wiedermann (2021), involves removing variables or specific effect vectors from log-linear models. If other effects lose statistical significance after this removal, they are interpreted as depending on, or being caused by, the omitted terms. Although this method can be used in both variable-oriented and person-oriented research, it is also not followed here because it does not permit direct testing of hypotheses about particular directed effects.

A fourth method for identifying the direction of causation centers on estimating reciprocal relationships, that is, situations where *X* influences *Y* and also *Y* influences *X* (*X* ⇆ *Y*). In this framework, the dominant direction is determined by comparing the size and statistical significance of the two paths, *X* → *Y* and *Y* → *X*. For example, when the coefficient for *X* → *Y* is significantly different from zero while the reverse path *Y* → *X* is not statistically significant, this suggests that is more likely that changes in *X* lead to changes in *Y*. Approaches for estimating reciprocal effects often rely on instrumental variables (e.g., James & Singh, 1978). However, such effects can also be estimated without instrumental variables by incorporating information from higher-order moments (cf. Ozaki & Ando, 2009). For categorical variables Wiedermann & von Eye (2020b) proposed event-based reciprocal log-linear models, which use Schuster-transformed non-orthogonal design matrices to estimate the targeted effects in both causal directions.

In this article, we continue the development of a fifth approach. This approach was first proposed by von Eye et al. (2026; cf. von Eye, & Wiedermann, 2026) in the context of a discussion of hypotheses that can be tested with Configural Frequency Analysis. The hypotheses that can be tested in this approach are new to CFA. The new approach allows researchers to specify hypotheses that involve a selection of cells only, and to test them in confirmatory CFA. In the present study, we use this new approach to test confirmatory hypotheses of person-oriented mediation.

## Relevance Logic and Implications

Coming back to Table 1, above, we now discuss what it is that is true or false in elementary and in composite propositions. Statement calculus is concerned with the truth of propositions, no matter their content. This can be seen most clearly in those cases of implication, in which the premise is false, but the composite proposition is true nevertheless. Consider the last two rows of the fifth column in Table 1. The entries in these cells indicate that the implication *X* → *Y* is true even if *X* is false. Accordingly, the second and the fourth entry in the last column of Table 1 indicates that the implication $\:X\to\:Y$is true whenever * X*is false, regardless of whether * Y*is true or false. Therefore, the implication can be expressed equivalently as $\:\neg\:(X\wedge\:\neg\:Y)$, where $\:\neg\:$ denotes negation. The same reasoning applies to the reverse implication $\:Y\to\:X$, which is equivalent to $\:\neg\:(Y\wedge\:\neg\:X)$.

In both of these cases, *Y* → *X* and *Y* → *X*, the rule applies that “ex falso sequitur quodlibet,” that is, from something false, anything follows. However, when the form, e.g., *X* → *Y*, is filled with substance, an implication that is formally true can intuitively be viewed, by many, as counterintuitive, paradoxical, even false, in particular when the implication arrow, →, is interpreted as in standard language use, that is, not as in classical logic. Most famous is the statement concerning the moon: “if the moon consists of green cheese, then …” For example: “if the moon consists of green cheese, then 2 + 2 = 5.” Formally, this composite proposition is true. However, this is the case only because the premise is false and, therefore, any implication that is based on this false premise will be true. This and other characteristics of logic gave rise to the well known paradoxes (see, e.g., Perelman, 1936; Slater, 2007) and contradictions of logic (Russell, 1908). In the present article, we are less concerned with the paradoxical nature of certain propositions. Rather, we ask questions concerning the link between the content of propositions and their truth, in mediation analysis.

Repeatedly, it has been attempted to use statement calculus and variants of it for the specification of hypotheses (e.g., Hildebrand, Laing, & Rosenthal, 1977; Mello, 2021; Ragin, 2008; von Eye & Brandtstädter, 1988). This, however, has proven problematic, for the following reasons. Statement calculus is abstract in the sense that it does not take into account features of objects that are examined. In other words, the truth of a proposition is determined solely based on form, *no matter the semantic content* (Russell, 1908), and it cannot be altered by empirical knowledge. The reason for this is that the semantic content of a proposition is not part of the decision concerning its truth (see, e.g., Beall, Restall. & Sagi, 2024). In addition, the rules of statement calculus do not add semantic content to propositions. Instead, they show ways to combine semantic content and to structure it. Thus, *formal elements* involve the rules of logic, and *material elements* are concerned with the semantic content. Material elements represent a priori knowledge and play no role when the truth of composite propositions is determined. Material elements exist a priori, or they can be generated by experience (observations, experiments, recordings).

What is needed is a general purpose logic, that is, an approach to logic that allows one to incorporate both formal and material elements. Such an approach can be seen in *relevance logic* (aka relevant logic; see Anderson, & Belnap, 1975; Anderson et al., 1992; Logan, 2024; Mares, 2024). In the paradoxes hinted at above and in many other paradoxes, the premises (antecedents) of implications seem to be irrelevant for the outcome (consequent). Consider the example discussed in the preceding section, the implication “if the moon consists of green cheese, then 2 + 2 = 5” is formally true, but so is “if the moon consists of green cheese, then 2 + 2 = 4.” In neither case, the premise has any relevant substantive connection to the outcome. Therefore, in relevance logic, it is required that the premise and the outcome share relevant content. This is termed the *variable sharing principle*. Standefer (2024, p. 1) defines this principle as follows: “*Variable-sharing criterion*: A logic *L* satisfies the *variable-sharing criterion* iff for any formulas *A*, *B*, if *A* → *B* is valid in *L*, then *A* and *B* share a propositional variable” (where ‘iff’ means ‘if and only if’). The important aspect of this principle is that variable-sharing adds some non-logical content. Therefore, implications are no longer true or false based on just the form of the proposition. Instead, relevant logic requires a substantive connection between premise and outcome. For an implication to be true, it must both be formally true and possess non-logical content that is shared by the antecedent and the outcome. Because of this, the paradoxes of implication that have been discussed solely in terms of classical logic must be rejected (the line of thought that concerns paradoxes is not pursued in this article any further; see, e.g., Standefer, 2024).

In the present context, two characteristics of this rejection are of note. The most important characteristic concerns the operator implication (→) specifically, the truth of implications in which the premise is false. As was illustrated in the last two columns of Table 1 and discussed above, an implication is true when both, the premise and the outcome are true, and whenever the premise is false. In the latter case, there can be no variable sharing. Therefore, from the perspective of a relevance logician, the implication cannot be true, and the arguments in which the premise is false are considered irrelevant. This can be seen as parallel to the well known *fallacy of the non sequitur*. Schagrin & Rescher (2026) state that this fallacy “occurs when there is not even a deceptively plausible appearance of valid reasoning, because there is an obvious lack of connection between the given premises and the conclusion drawn from them.” The arguments in which the premise is true can be relevant, but they must exhibit variable sharing.

The second characteristic is that relevance logic does not provide rules that specify variable sharing. Most authors state that variable sharing is based on a priori developed substantive theories that are not part of relevance logic. This lack of rules concerning variable sharing is what makes relevance logic a general purpose logic. Any science in which implications are considered can propose theories that enable variable sharing. The truth of implications rests, then, on logic (both, the premise and the outcome must be true) as well as on the substantive theory in which premise and outcome are related by way of variable sharing.

## Mediation Analysis and Relevance Logic

Figure 1 displays the flow of information as it usually is conceptualized in the social and behavioral sciences when mediation is posited. The paths *a*, *b*, and *c* are, in variable-oriented analysis, represented by regression coefficients (continuous variables) or logits (categorical variables). In person-oriented analysis, these paths are represented by the co-occurrence (or lack of it) of variable categories that is observed significantly more often (or less often) than expected under a suitable base model.

Before moving to the application of CFA to mediation hypotheses, we link the threads of relevance logic and mediation. We ask, how the model of mediation depicted in Fig. 1 can be represented using the tools of statement calculus and relevance logic. The truth values for the elements of mediation are given in Table 3.

**Table 3:** Truth table for elements of mediation analysis (T = True, F = False, *i* = irrelevant)

Truth values of individual variables			Truth values for elements of mediation					
*X*	*M*	*Y*	*X* → *M*		*M* → *Y*		*X* → *Y*	
T	T	T	T		T		T	
T	T	F	T		F		F	
T	F	T	F		T	*i*	T	
T	F	F	F		T	*i*	F	
F	T	T	T	*i*	T		T	*i*
F	T	F	T	*i*	F		T	*i*
F	F	T	T	*i*	T	*i*	T	*i*
F	F	F	T	*i*	T	*i*	T	*i*

In the first three columns of Table 3, the truth values of the three variables are given that are involved in the mediation model. These variables are the Predictor, *X*, the Mediator, *M*, and the outcome, *Y*. As in Table 2, the three binary variables are crossed, so that every combination of truth values becomes possible. In the fourth column, the truth values for the implication are given that are depicted by Path *a* in Fig. 1, that is, the path from predictor to mediator. To determine these values, we use classical statement calculus. As was described above, this implication is true when both, the predictor (premise) and the mediator are true, and whenever the predictor is false. In the sixth column of Table 3, the truth values for the implication are given that are depicted by Path *b* in Fig. 1, that is, the path from mediator to outcome. The truth values are determined just as in Column 4. The same applies to the truth values in Column 8. In this column, the truth values for the implication are given that are depicted by Path *c* in Fig. 1, that is, the path from predictor to outcome.

Table 3 also contains three columns (5, 7, and 9) in which *irrelevant cells* are labeled. These cells are considered irrelevant because the predictor is false (see Elkind, 2021; cf. vacuously true statements or counterpossibles; see Kocurek, 2021). They are not used for the decision whether mediation exists. However, they are not useless (more detail follows below).

The three columns 4, 6, and 8 represent the truth values of the three individual implications involved in the mediation model in Fig. 1. The truth values for the combined paths from the predictor to the mediator and from the mediator to the outcome appear in Table 4.

Table 4 presents relevance logic definitions full and partial mediation mechanisms and illustrates the distinction between conjunctive and implicative mediation. Table 5 displays the corresponding truth values.

**Table 4:** Relevance logic definitions for conjunctive and implicative mediation mechanisms

Form of mediation	Mediation mechanism	Relevance logic definition
Conjunctive	Full Mediation	(*X* → *M*) ∧ (*M* → *Y*)
	Partial Mediation	{(*X* → *M*) ∧ (*M* → *Y*)} ∧ (*X* → *Y*)
Implicative	Full Mediation	(*X* → *M*) → (*M* → *Y*)
	Partial Mediation	{(*X* → *M*) → (*M* → *Y*)} ∧ (*X* → *Y*)

**Table 5:** Truth values for conjunctive and implicative forms of mediation (T = True, F = False, *i* = irrelevant)

Conjunctive mediation					Implicative mediation			
Full mediation		Partial mediation			Full mediation		Partial mediation	
T		T			T		T	
F		F			F		F	
F	*i*	F		*i*	T	*i*	F	*i*
F	*i*	F		*i*	T	*i*	F	*i*
T	*i*	T		*i*	T	*i*	T	*i*
F	*i*	F		*i*	F	*i*	F	*i*
T	*i*	T		*i*	T	*i*	T	*i*
T	*i*	T		*i*	T	*i*	T	*i*

Using Tables 4 and 5, we now introduce four forms of mediation. Based on the truth values in Table 3, one can uniquely describe these four forms. The first two, called *conjunctive full mediation* and *conjunctive partial mediation*, link the implications from premise to mediator, from mediator to outcome, and from premise to outcome, that is, Paths *a*, *b*, and *c*, by way of conjunction. The truth values for these two forms of mediation appear in Columns 1 and 3 of Table 4.

The other two, called *implicative full* and *implicative partial mediation*, link the implications that are represented by Paths *a*, *b*, and *c* by way of implication. The truth values of these two forms appear in Columns 5 and 7 in Table 5. The irrelevant cells of the four forms of mediation appear in Columns 2, 4, 6, and 8. Here, cells are labeled as irrelevant when they are irrelevant in one of the implications in Table 3. That is, whenever a cell is irrelevant, it cannot change back to being ‘not irrelevant.’

Evidently, conjunctive full and partial mediation as well as implicative full and partial mediation all require that the premise, the mediator, and the outcome all be true. When the outcome variable is false, all four forms of mediation are false. To distinguish between the four forms, we need to use the irrelevant cells (marked by *i* in Tables 3 and 5). These cells are not used when it comes to determine whether mediation exists. However, they can be of use for the distinction between the four forms.

First, we compare the truth values of implicative full and partial mediation (Columns 5 and 7 in Table 5). These two forms differ in the third and the fourth row in Table 5. These are the configurations T – F – T and T – F – F. Full implicative mediation can be true when the predictor is true, the mediator is false, and the outcome is true. Partial implicative implication can exist when the outcome is false as well. Still, it should be realized that these patterns are irrelevant because the mediator is false.

In contrast, the two forms of conjunctive mediation (Table 4 and Columns 1 and 3 in Table 5) cannot be distinguished even based on the irrelevant cells. The reason for this is that, when the expression (*X* → *M*) ∧ (*M* → *Y*) is true, adding the term (*X* → *Y*) by using the conjunction operator does not change the truth of the composite. That is, the composite ((*X* → *M*) ∧ (*M* → *Y*) ∧ (*X* → *Y*) is true when (*X* → *M*) ∧ (*M* → *Y*) is true (cf. the conjunctive syllogism discussed by Standefer, 2024). We now move to the statistical analysis of mediation with CFA. We ask how mediation can be analyzed with CFA.

## Mediation and CFA

In this article, we propose using implications to represent the relations depicted by the paths *a*, *b*, and *c*, in Fig. 1. Specifically, we propose interpreting Path *a* as ‘the predictor (premise) implies the mediator,’ Path *b* as ‘the mediator implies the outcome,’ and Path *c* as ‘the predictor (premise) implies the outcome.’ Second, we propose using the implicative form of mediation, because the conjunctive form does not allow one to distinguish between full and partial mediation. In the implicative form, the original implications *a* and *b* are related to each other by way of implication. Third, we propose using CFA for analysis of these propositions.

In exploratory CFA, tests are performed for each pattern of variable categories, that is, for each configuration, to determine whether more or fewer cases exhibit this pattern than expected. When there are significantly more cases, a configuration is said to constitute a *CFA type*. When there are significantly fewer cases, a configuration is said to constitute a *CFA antitype*. The expected frequencies are estimated under a base model. This model contains all effects that are not of interest to the researcher. When the model is rejected, types and antitypes indicate where in the cross-classification of the variables effects of interest, that is, *local effects*, exist. In confirmatory CFA, tests are performed for a selection of cells.

In both approaches to CFA, the nominal significance level α is protected with reference to the number of tests performed. In confirmatory CFA, this number is smaller than in exploratory CFA. Therefore, they have higher statistical power.

In the present context of confirmatory CFA of hypotheses of mediation, we focus on configurations that are relevant (see Tables 3 and 4). In addition, we focus on those relations that are indicative of full and of partial implicative mediation. To explain, we use Table 5. Columns 5 and 7 contain the relations of interest. The first of these, that is, Column 5 in the table, displays the truth values for full implicative mediation. The first two configurations represent this model of mediation, all others are irrelevant. The first is the configuration for which all three variables, that is, the predictor, the mediator, and the outcome are true (see the first three columns of Table 3). The second configuration is the one for which the outcome is false. In the remaining configurations, the predictor, the mediator, or both are false. The hypothesis of full implicative mediation is confirmed when the first configuration constitutes a type.

The relation between the predictor and the outcome is represented in the last two columns of Table 3. The mediator is, in this relation, not considered. The first and the third configurations are those for which both, the predictor and the outcome are true. If both of these constitute types, the hypothesis of a direct predictor – outcome relation is confirmed.

In CFA, it is most important to specify a suitable base model. To test the hypothesis of full mediation, the base model posits that the predictor, the mediator, and the outcome are independent. The model is, therefore, $\:\mathrm{log}\widehat{m}=\lambda\:+{\lambda\:}^{X}+{\lambda\:}^{M}+{\lambda\:}^{Y}$. This model suggests that the three variables are completely independent. None of the pairwise relations is included. When this model is rejected, one or more of the pairwise relations, they are hypothesized in the paths *a*, *b*, and *c*, are bound to exist.

As far as the three-way interaction, *X* × *M* × *Y*, is concerned, there are two options. The first involves following the principle of parsimony (Wu & Hamada, 2000; von Eye et al., 2009). According to this principle, the important effects are carried by lower order terms. Higher order terms carry only little weight, if any. Under this assumption, the three-way interaction can be omitted from the base model when mediation hypotheses are tested. When, however, the principle of parsimony is not applied, the three-way interaction can be included, and the model becomes non-hierarchical, specifically $\:\mathrm{log}\widehat{m}=\lambda\:+{\lambda\:}^{X}+{\lambda\:}^{M}+{\lambda\:}^{Y}+{\lambda\:}^{XMY}$. The configurations of interest are the same under both base models. To repeat, in full mediation, only the paths from the premise to the mediator and from the mediator to the outcome exist. In partial mediation, the path from the premise to the outcome exists as well.

## Data example

In the following example, we resume the analysis of data from the Overcoming The Odds (OTO) study (Lerner et al., 2002; Taylor et al., 2002; for an exploratory mediation analysis of these data, see von Eye et al., 2009). The OTO is a longitudinal study of the role that individual and ecological assets play in the development of adolescent African American male youth. The participants in this study were either gang members (*n* = 45; average age at the beginning of the study = 15.82 yrs) or members of community-based organizations (CBO; *n* = 50; average age at the beginning of the study = 16.31 yrs). The participants indicated whether they had been sexually active in the year before the interview. The responses were scored as 1 = sexually active and 2 = not active, and 1 = gang member; 2 = CBO member. In the following analyses, we test the implicative mediation hypothesis that the link between sexual activity reported in the first interview (Sex1) and sexual activity reported in the second interview (Sex2) is mediated by group membership (Gang or CBO member). In contrast to the earlier analyses the present ones are confirmatory in the sense that we entertain the hypothesis that sexual activity over the interview period is fully mediated by gang membership.

The CFA of the Sex1 × Gang × Sex2 cross-classification is set up as follows. First, we proceed under the principle of parsimony and specify the base model of variable independence, $\:\mathrm{log}\widehat{m}=\lambda\:+{\lambda\:}^{Sex1}+{\lambda\:}^{Gang}+{\lambda\:}^{Sex2}$. To test the hypothesis of implicative full mediation, we protect α using the Bonferroni procedure. This results in the protected α* = 0.05/2 = 0.025. We apply CFA tests only to Cell 1 1 1 and Cell 1 1 2. We use the *z*-test. First, however, we perform standard CFA. Table 6 summarizes the result of a standard first order CFA, that is, a CFA of variable independence. This CFA is performed with respect to a different question, that of variable independence. Therefore, for this CFA, we protect α as in standard exploratory CFA, that is, with respect to all possible CFA tests. The Bonferroni-protected α* for this analysis is α* = 0.05/8 = 0.00625.

**Table 6:** First Order CFA of the Cross-classification of Sex1 (S1), Gang (G), and Sex2 (S2) (n.s. = not significant, *i* = irrelevant for implicative full mediation)

S1 G S2	m	$\:\widehat m$	z	*p*	Decision
1 1 1	21	3.63	9.117	0.0000	Type
1 1 2	2	9.63	−2.459	0.0070	Antitype
1 2 1	4	4.03	−0.017	0.4934	n.s.
1 2 2	1	10.70	−2.966	0.0015	Antitype (*i*)
2 1 1	0	8.69	−2.947	0.0016	Antitype (*i*)
2 1 2	22	23.05	−0.219	0.4134	n.s.
2 2 1	1	9.65	−2.785	0.0027	Antitype (*i*)
2 2 2	44	25.61	3.633	0.0001	Type (*i*)

The overall goodness-of-fit for this base model is LR-χ^2^ = 103.66. For *df* = 4 and *p* < 0.001, this model is rejected. For the exploratory CFA, two types and four antitypes emerge. The first type, constituted by Cell 1 1 1, suggests that more adolescent gang members than expected under the base model had been sexually active in the years before the two interviews. The second type, constituted by Cell 2 2 2, indicates that more adolescents that were CBO members reported that they had not been sexually active during that year.

The four antitypes suggest that the following patterns are particularly unlikely:

1. change from sexually active to inactive in gang members (Configuration 1 1 2).
2. change from sexually active to inactive in CBO members (Configuration 1 2 2);
3. change from sexually inactive to active in gang members (Configuration 2 1 1); and.
4. change from sexually inactive to active in CBO members (Configuration 2 2 1).

We now ask whether the relevance logic-based hypothesis can be retained according to which sexual activity during the span covered by the interview is mediated by gang membership. According to Table 6, a type constituted by Configuration 1 1 1 would be in support of this hypothesis, but only if Configuration 1 1 2 does not also constitute a type. Table 6 shows that this is indeed the case. In fact, Configuration 1 1 2 constitutes an antitype, suggesting that fewer adolescents that were gang members reported that they were sexually active in the time before the first interview and sexually inactive before the second interview. Here, conclusions concerning the Configurations 1 1 1 and 1 1 2 hold, regardless of the adjusted alpha level (α* = 0.025 for testing the hypothesis of implicative full mediation and α* = 0.00625 for the exploratory analysis of the full cross-table). All other configurations are irrelevant and do not speak to the relevance logic-based mediation hypothesis. Table 6 shows that, in addition to the type that is constituted by Configuration 1 1 1 and the antitype that is constituted by Configuration 1 1 2, three additional antitypes and one additional type emerge. These, however, only speak the hypothesis of variable independence. They indicate, where in the cross-classification, the base model hypothesis is violated, with no reference to the mediation hypothesis.

Table 6 also shows how to distinguish between full and partial implicative mediation. The type that is constituted by Configuration 1 1 1, supports a hypothesis of implicative mediation. To distinguish between full and partial mediation, we inspect Configurations 1 2 1 and 1 2 2. To support partial implicative mediation, Configuration 1 2 1 must also constitute a type while Configuration 1 2 2 must either not deviate from expectation or even constitute an antitype. In the data example in Table 6, only the latter is the case, the former is not. Therefore, we conclude that, in the present example, full mediation prevails.

In the context of relevance logic, it is important to make explicit the variable that is shared in the three implications in Paths *a*, *b*, and *c*. In the present example, this variable is Group Membership. In each of the three paths, Group Membership is considered closely related to sexual activity. Specifically, Group Membership is predictive of sexual activity of gang members as it is reported in the two interviews. This applies even in Path *c* where group Membership is not explicitly involved.

It is important to realize that this result contradicts the ones presented by von Eye et al. (2009) (cf. Smyth & MacKinnon, 2020; Wiedermann, & von Eye, 2021). In these analyses, partial mediation was supported. The reason for these differences is that, to obtain the earlier results, information was used, that, here, is considered irrelevant. Patterns of types and antitypes are examined and interpreted that, although they exist, are of no relevance for the person-oriented, relevance logic-based hypothesis of implicative mediation that is tested here. In the present context, Configurations 1 2 1 and 1 2 2 are used only to come to a decision concerning the existence of full versus partial implicative mediation.

It is also important to realize that mediation analysis at the level of individual configurations opens the doors for hypotheses that differ across configurations, in the same data. In other words, different groups of individuals can be hypothesized to travel different pathways. Consider again the data example in Table 6. In this example, gang members were hypothesized to report having been sexually active in the time periods before both interviews. That is, the hypothesis was that gang membership mediates these two reports. This hypothesis was confirmed. Full mediation exists.

Clearly, this hypothesis does not involve CBO members. For these respondents, we now hypothesize a different path of mediation. Specifically, we hypothesize that CBO membership mediates reporting of having not been sexually active in the periods before the two interviews. Configuration 2 2 2 supports this hypothesis. Indeed, this configuration constitutes a CFA type. To be interpretable as also supporting the hypothesis that CBO membership is a mediator between these two reports, Configuration 2 2 1 must either constitute an antitype or must be conform with the hypothesis of variable independence. The antitype in Table 5 supports the mediation hypothesis. To support a hypothesis of implicative partial mediation, Configuration 2 1 2 must also constitute a type and Configuration 2 1 1 must either constitute an antitype or conform to the base model. Configuration 2 1 2 does not constitute a type. Therefore, the hypothesis of partial mediation is rejected. Implicative full mediation prevails.

In the final step of this analysis, we ask whether proceeding under the principle of parsimony is justified, for the present data. To answer this question, we estimate a log-linear model that includes all possible two-way interactions, that is, the model $$\:\log\widehat m=\lambda\:+{\lambda\:}^{Sex1}+{\lambda\:}^{Gang}+{\lambda\:}^{Sex2}$$$$+{\lambda\:}^{Sex1,Gang}+{\lambda\:}^{Sex1,Sex2}+{\lambda\:}^{Gang,Sex2}$$. This model describes the frequency distribution very well (LR-χ^2^ = 1.25; *df* = 1; *p* = 0.264). This LR- χ^2^ is so small that it is impossible for the three-way interaction to explain a significant portion of variability. This is also confirmed when inspecting Bayes Information Criteria (BICs) for the two-way interaction model and the model that, in addition, incorporates the three-way interaction. Despite the fact that the three-way interaction model is saturated and, therefore, perfectly reproduces observed frequencies, the two-way interaction model shows the smaller BIC (BIC_two−way_ = 41.14, BIC_three−way_ = 41.97). Therefore, this interaction cannot be the cause for a configuration to constitute a type or an antitype. We, therefore, conclude that it can be justified to proceed under the principle of parsimony, because the three-way interaction cannot be the cause for Type 1 1 1 to emerge.

## Discussion

When applying statement calculus to evaluate substantive propositions, results can emerge that suggest implausible variable relations. This applies in particular when these relations involve implications (von Eye, & Wiedermann, 2026) or mediation processes, that is, processes that are not commutative. One reason why implausible results can emerge is the well known ’ex falso sequitur quodlibet’ rule, that is, from something false, anything can follow. In mediation analysis, this rule indicates that when the premise is not observed, or even false, the outcome is always true. Repeated attempts to apply statement calculus to substantive statements have proven futile, for this reason.

Relevance logic breaks with this rule. It requires that the premise be true. Only then, and when the outcome is true as well, an implication can be true. In addition, relevance log requires that the premise and the outcome exhibit variable sharing, that is, share substantive meaning for an implication to be true.

These two characteristics of relevance logic have two consequences. First, there will be no implausible or subjectively untrue implications when relevance logic is applied in substantive research. In the analysis of direction of causation and in mediation analysis, results become, therefore, more credible. Second, the number of patterns is dramatically reduced that can be used to support direction dependence or mediation hypotheses. Therefore, the cell-wise CFA tests have more power than the tests in standard exploratory CFA. Consider, for example, Table 5, above. Only two of the eight cells of the cross-classification of the premise, mediator, and outcome are of relevance for the decision as to whether mediation exists. The remaining eight are (1) either not used for this decision, (2) used only to distinguish between full and partial mediation, but (3) used for the estimation of the expected cell frequencies.

Using (relevance) logic in substantive mediation analysis has one additional important consequence. It allows one to distinguish between conjunctive and implicative models of mediation. In the former, the model links the individual implications by way of conjunctions: For full mediation, Path *a* must exist *and* Path *b* must exist as well. For partial mediation, Path *c* must exist in addition. Interestingly, relevance logic does not allow one to distinguish between full and partial conjunctive mediation.

In contrast, this distinction is possible when the individual implications are linked by way of implications: For full mediation, Path *a* implies Path *b*. For partial mediation Path *c* must exist as well (conjunction). Here, patterns appear that differ in full from partial mediation (see Table 4). This applies accordingly when multiple predictor, mediator, and outcome variables are used.

This last issue leads to a very important but as of yet not adequately discussed element of research methods in person-oriented mediation analysis: the characteristics of the flow of information. In the analysis of metric variables, briefly reviewed at the beginning of this article, the exclusive focus is on regression-type relations. Here, the decision becomes possible and – in empirical research – necessary whether Paths *a* and *b* must both exist without *b* being dependent upon or implied by *a*. When a relation between Paths *a* and *b* exists, models of moderated mediation (i.e., models that include the predictor × mediator interaction in the outcome model) can be entertained. In the person-oriented mediation context, the present approach allows one to discuss relations not only between variables but also relations among relations, that is, here, relations between two implications. In either case, Path *c* is added, in partial mediation, by way of conjunction. This is necessary for the distinction between full and partial mediation to be identifiable. The development of person-oriented moderated mediation models constitutes an important direction for future research.

## Data Availability

The presented statistical analysis uses categorical data. The corresponding cross table is presented in the article.
